# The value of basement membrane-associated genes in the prognosis and immune regulation of glioma

**DOI:** 10.1097/MD.0000000000033935

**Published:** 2023-06-09

**Authors:** Yanqi Sun, Ren Li, Yang Chen, Biao Yang, Xuepeng Li, Ziao Li, Jianhang He, Zihan Zhou, Jiayu Li, Xiaolong Guo, Xiaogang Wang, Yongqiang Wu, Wenju Zhang, Geng Guo

**Affiliations:** a Department of Emergency, The First Hospital of Shanxi Medical University, Taiyuan, Shanxi, China; b Department of Neurosurgery, The First Hospital of Shanxi Medical University, Taiyuan, Shanxi, China.

**Keywords:** basement membrane, clinical, glioma, immune, prognosis

## Abstract

Gliomas have a high incidence rate in central nervous tumors. Although many breakthroughs have been made in the pathogenesis and treatment of glioma, the recurrence and metastasis rates of patients have not been improved based on the uniqueness of glioma. Glioma destroys the surrounding basement membrane (BM), leading to local infiltration, resulting in the corresponding clinical and neurological symptoms. Therefore, exploring the biological roles played by BM associated genes in glioma is particularly necessary for a comprehensive understanding of the biological processes of glioma and its treatment. Differential expression and univariate COX regression analyses were used to identify the basement membrane genes (BMGs) to be included in the model. LASSO regression was used to construct the BMG model. The Kaplan–Meier (KM) survival analysis model was used to assess the prognosis discrimination between training sets, validation sets, and clinical subgroups. Receiver-operating characteristic (ROC) analysis was used to test the prognostic efficacy of the model. Use calibration curves to verify the accuracy of nomograms. Gene ontology (GO), Kyoto encyclopedia of genes and genomes (KEGG), and gene set enrichment analysis (GSEA) were used to analyze the function and pathway enrichment among the model groups. ESTIMATE and other 7 algorithms including CIBERSORT were used to evaluate the immune microenvironment. “pRRophetic” was used to evaluate drug sensitivity. This study demonstrated that high-risk genes (LAMB4, MMP1, MMP7) promote glioma progression and negatively correlate with patient prognosis. In the tumor microenvironment (TME), high-risk genes have increased scores of macrophages, neutrophils, immune checkpoints, chemokines, and chemokine receptors. This study suggests that BMGs, especially high-risk-related genes, are potential sites for glioma therapy, a new prospect for comprehensively understanding the molecular mechanism of glioma.

## 1. Introduction

Gliomas have a high incidence rate in the human brain. Because of its strong drug resistance and immune escape, glioma patients have a low survival rate and a short median survival period.^[1]^ The current clinical consensus is that surgical resection combined with postoperative chemoradiotherapy does not significantly improve the survival rate of patients. As a biological barrier, the blood-brain barrier (BBB) could protect nerve tissue and resist the invasion of viruses and bacteria in the central system. During the treatment of glioma, the BBB can prevent the entry of chemotherapeutic drugs, which leads to the failure of the drugs.^[2]^ It is known that the BBB is mainly composed of vascular endothelial cells and basement membranes (BM).^[3]^ The BM supports the integrity of the BBB indispensably. The vascular BM participates in the integration of nerve cells.

BM is a kind of unique extracellular matrix of animal cells, which participates in human body reactions such as cell tissue adhesion and cell polymerization.^[3]^ The BM is composed of extracellular matrix and other components, and Laminin as an important component in the extracellular matrix can connect the components between cells to form the complete cytoskeleton.^[4]^ Mutations or deletions of basement membrane genes (BMGs) can lead to related diseases such as congenital muscular dystrophy, congenital nephropathy, and cataract.^[5–8]^ The components of BM, such as Laminin, integrin, and collagen, participate in the metastasis process of tumor cells, which aggravates the malignant degree of the tumor. It has been shown that genes related to the BM promote invasion and infiltration and accelerate the malignant progression of glioma.^[9]^ Therefore, it is necessary to further clarify the role of BM and BMG in promoting the malignant transformation of glioma and explore effective clinical treatment strategies.

According to this study, we verified the relationship between BMG and survival time and survival risk of glioma patients. Furthermore, the immune-related targets of BMGS were analyzed, and this study shows that the expression of BMGs in high-risk populations will accelerate the malignant transformation of glioma patients. We demonstrated that BMGs may be used as a target for glioma therapy and provide a new target for molecular targeted therapy of glioma.

## 2. Materials and methods

### 2.1. Data preparation

To analyze differences in BMGs expression in normal and glioma samples, we retrieved transcriptional profiling for 689 The Cancer Genome Atlas (TCGA) glioma cases and 1137 Genotype-Tissue Expression (GTEx) normal samples from the UCSC Xena database (https://xenabrowser.net/datapages/).^[10]^ All samples were processed by the Toil process.^[11]^ The transcriptional profiling, clinical data, and 667 glioma samples used for subsequent analyses were from the TCGA (https://portal.gdc.cancer.gov/).^[12]^ We retained TCGA glioma samples with complete survival data and clinical features by extracting the transcriptome and clinical data using the R software. RNAseq data from 276 glioma samples from the GEO were used for the test set (accession number: GSE16011; https://www.ncbi.nlm.nih.gov/geo/query/acc.cgi?acc=GSE16011). Clinical data of samples was derived from the original literature of GSE16011.^[13]^ The CGGA database was used to obtain the RNAseq and clinical information for the 693 and 325 glioma samples (DataSet ID: mRNAseq_693, mRNAseq_325; http://www.cgga.org.cn/download.jsp) as the additional test sets.^[14–18]^ We extracted consensus gene expression matrices for the 4 datasets from TCGA, GEO, and CGGA, respectively. To model and remove data noise and obtain correct statistical inferences, the 4 datasets were calibrated using the “sva” R package.^[19]^

### 2.2. Differential analysis of BMGs and construction of the BMGs risk model

BMGs differential expression analyses between normal and glioma samples were conducted via the R package “limma.”^[20]^ The differential expression threshold was set to FDR < 0.05 and | logFC | > 1. The “ggplot2” R software allowed for the completion of the volcanic map. The heatmap of differential genes was visualized using the R package “pheatmap.” To find BMGs associated with prognosis, a single-variable COX regression analysis was utilized. The optimal model was fitted using LASSO regression analysis via the R package “glmnet.”^[21]^ Making use of the penalty parameter calculated via 10-fold cross-validation, a criterion of optimality and minimum penalty (λ) was chosen to avoid overfitting. The multivariate stepwise COX regression analysis we used to get patient risk scores. The formula for calculating risk score:


$$Risk\;score=\sum_{i}coeffient\left(BMGs_{i}\right)*Expression\left(BMGs_{i}\right)$$

The coefficient and expression in the formula denote the regression coefficient of each BMG and the normalized expression for each BMG. We divided the train and test sets samples into high- and low-risk cohorts based on the median of the samples’ risk scores for the ensuing analyses.

### 2.3. Prognostic analysis and validation of the BMGs risk model

We performed risk profile analyses of overall survival (OS) through the high- and low-risk cohorts in train and test sets via the R package “survminer.” In the train set and the test sets, we used risk scatter plots to confirm the model capacity for surviving state discrimination. In the train and validation sets, the prognostic value of the model was evaluated using Kaplan–Meier (KM) survival analysis. Risk grouping and survival grouping information were presented in the KM survival curve. Receiver-operating characteristic (ROC) analysis was carried out on both the training set and test sets using the R package “timeROC” to assess the model capacity for prediction. The model genes heatmap was completed by R package “pheatmap.” To find independent prognostic determinants of OS in gliomas, multivariate COX analysis regression was employed.

### 2.4. Stratified analysis of clinical traits in the BMGs risk model

We performed the heatmap of stratification of clinical traits and risk, showing the difference in clinical traits between high-risk and low-risk cohorts. The box plots showing clinical traits differences between high-and low-risk cohorts were created using the R package “ggplot2.” According to the clinical trait subtypes, we performed KM survival curves for each subtype.

### 2.5. Performing nomogram and function enrichment analyses

We included age, sex, WHO grades, and risk score in fitting nomogram. Using the R package “regplot,” we conducted the nomogram. The bootstrapping method was used and repeated 1000 times to validate the nomogram. The calibration curve showed the nomogram prediction accuracy over 1, 3, and 5 years. The R package “rms” was used for all validation. With R packages “enrichplot,” “org.Hs.e.g..db” and “clusterProfiler,” we performed Gene Ontology (GO), Kyoto Encyclopedia of Genes and Genomes (KEGG), and Gene Set Enrichment Analysis (GSEA) on the differential genes.^[22–24]^ The threshold for differential expression in GO and KEGG was set to FDR < 0.05 and | logFC | > 1. All differential genes were included in GSEA. Statistical significance was defined as *P* value < .05.

### 2.6. Tumor immune microenvironment and drug sensitivity prediction analyses

The immune cell scores of all samples were calculated by 7 algorithms, including “TIMER,” “XCELL,” “CIBERORT,” “CIBERORT-ABS,” “EPIC,” “QUANTISEQ,” and “MCPCOUNTER.”^[25–30]^ The immune cell scoring results of the above 7 algorithms are reflected in the immune heatmap through the R package “pheatmap.” Distinctions in immune cell scores calculated by “TIMER,” “EPIC,” and “CIBERORT” between high-and low-risk were shown in boxplots. The difference in immune microenvironment between high-risk and low-risk cohorts was assessed using the R package “estimation.” Using R, we compared expression levels of the checkpoint, MHC molecule, chemokine, and chemokine receptor between high-risk and low-risk cohorts. The checkpoint and MHC molecule genes were acquired from the Charoentong research.^[31]^ The drug sensitivity analysis was carried out using the R package “pRRophetic.”^[32]^

### 2.7. Statistical analysis

All data are analyzed using R software (Version 4.2.1). The Wilcoxon rank-sum test was used to evaluate the difference analysis between 2 groups of quantitative data. The Spearman method was used for performing correlation analyses and calculating correlation coefficients. Utilizing the Log-rank test, statistical tests for survival analysis were conducted. Furthermore, *P* value < .05 was regarded as statistically significant.

## 3. Result

### 3.1. Differential analysis of BMGs and fitting of the BMGs risk model

**Figure 1.**
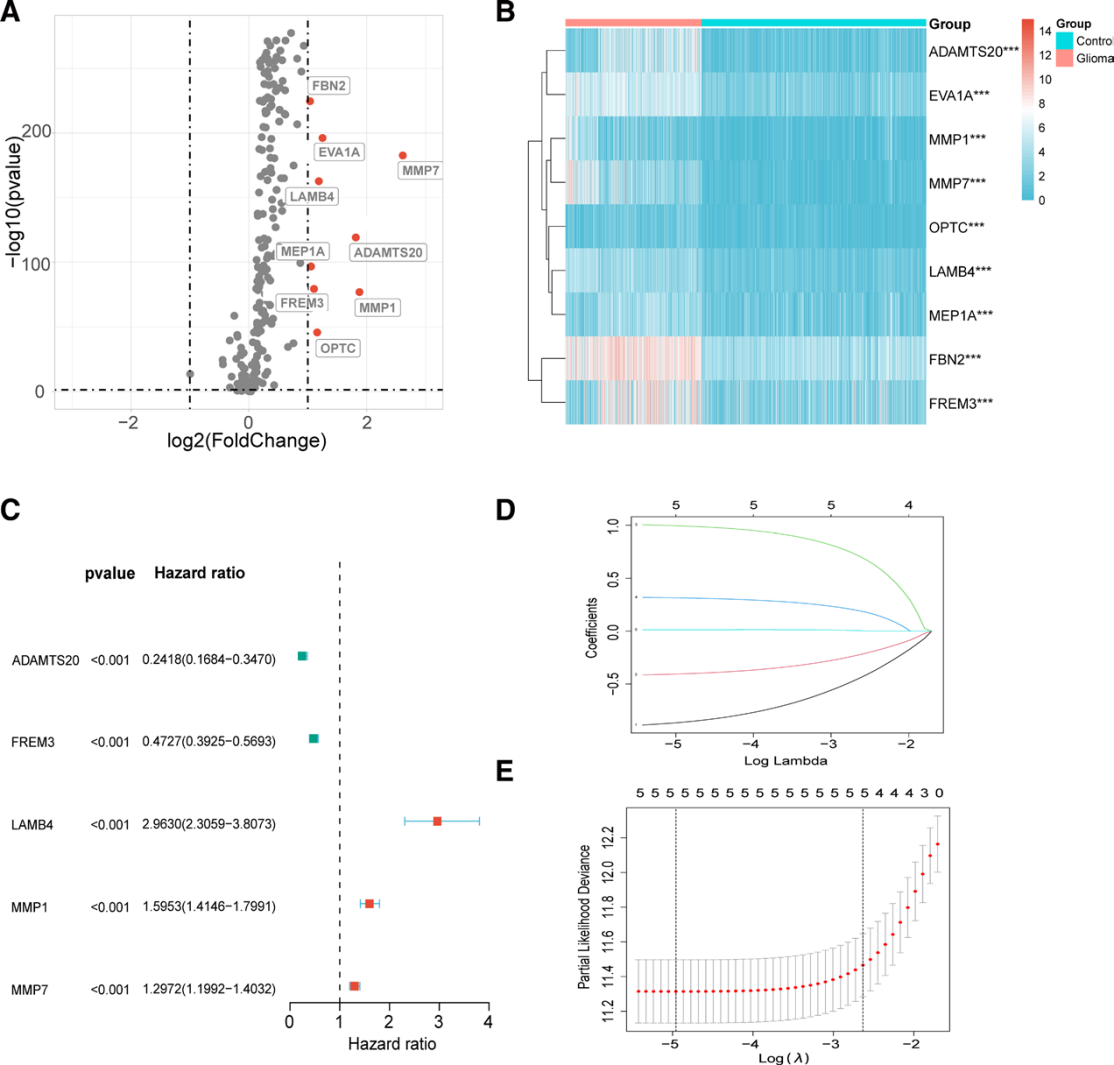
Differential analysis of BMGs and construction of the BMGs risk model. (A) Volcano plot of difference analysis. Red means up-regulated. (B) Heatmap of differential expression of BMGs in GTEx normal samples and TCGA glioma samples. *P < .05, **P < .01, ***P < .001. Red means up-regulated and blue means down-regulated. (C) Forest plot of univariate Cox regression analysis based on differential expressed BMGs. Red represents a high hazard ratio, green represents a low hazard ratio, and blue represents 95% CI of the hazard ratio. (D and E) LASSO regression analysis identified 5 BMGs for inclusion in the prognostic model for glioma patients. *P* value < .05 was deemed to be statistically significant. BMGs = basement membrane genes, GTEx = Genotype-Tissue Expression, TCGA = The Cancer Genome Atlas.

**Figure 2.**
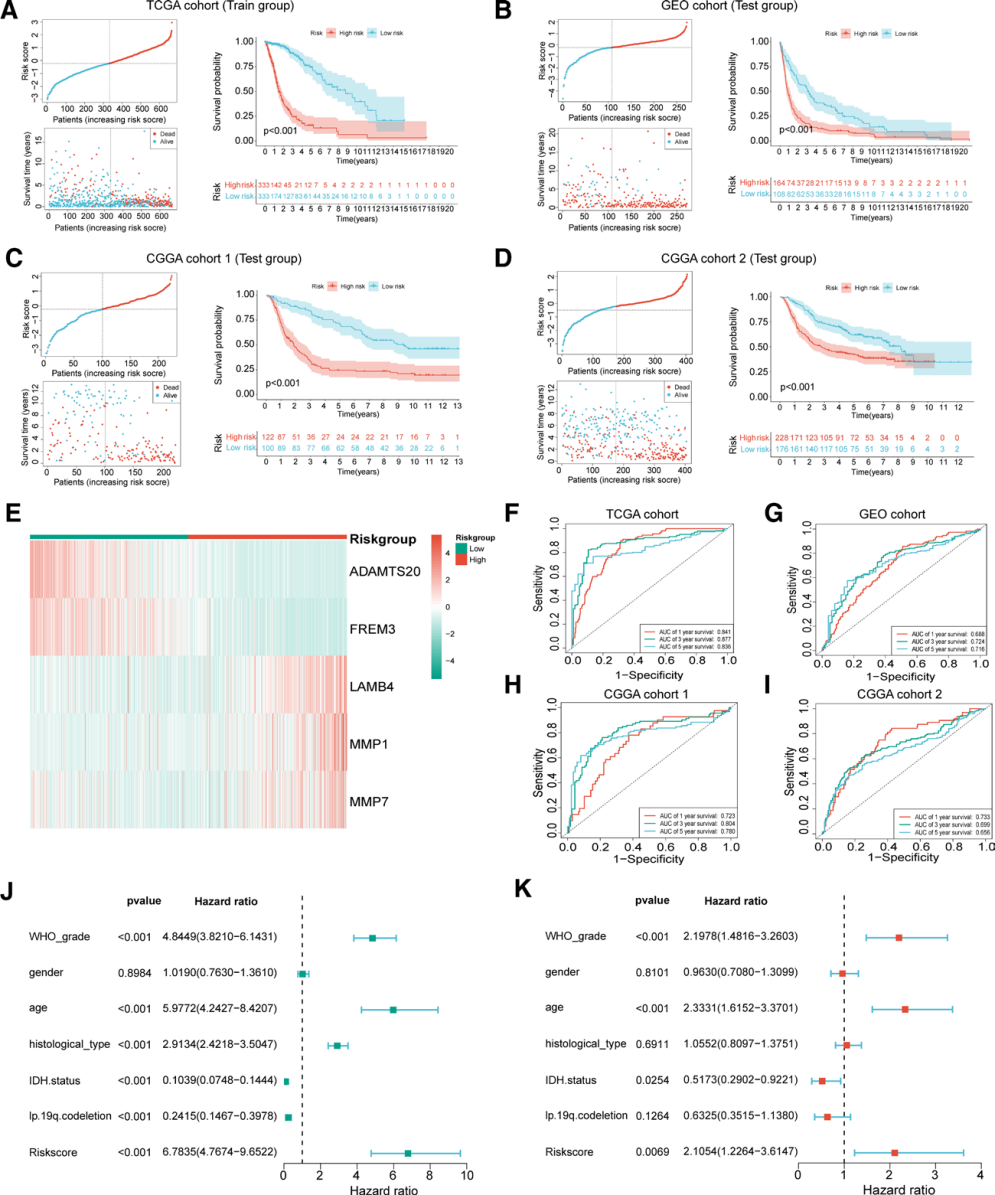
Prognostic analysis and validation of the BMG risk model. (A–D) Survival time and risk score, between the low-risk and high-risk groups in TCGA cohort (A), GEO cohort (B), CGGA_325 cohort (C), and CGGA_693 cohort (D). Red represents high risk and the green represents low risk. (E) Differential heatmap of model genes expression in high-and low-risk groups. Red represents high expression and green represents low expression. (F–I) ROC curves of TCGA cohort (F), GEO cohort (G), CGGA_325 cohort (H), and CGGA_693 cohort (I) at 1, 3, and 5 yr. Red represents 1-yr, green represents 3 yr and blue represents 5 yr. (J) Univariate Cox regression forest plot of clinical features and risk scores. (K) Multivariate Cox regression forest plot of clinical features and risk scores. *P* value < .05 was deemed to be statistically significant. TCGA = The Cancer Genome Atlas.

We performed differential expression analysis of 224 BMGs in GTEx normal samples and TCGA glioma samples and found 9 BMGs were significantly highly expressed in glioma samples (Fig. 1A). The differentially expressed genes (DEGs) are ADAMTS20, EVA1A, FBN2, FREM3, LAMB4, MEP1A, MMP1, MMP7, and OPTC. The heatmap showed the difference in DEGs expression between normal and glioma samples (Fig. 1B). “***” represented *P* value < .001. We obtained 5 BMGs associated with glioma prognosis by univariate COX regression analysis (Fig. 1C). They were ADAMTS20, FREM3, LAMB4, MMP1, and MMP7. The LASSO regression included all 5 BMGs with prognostic significance to fit the BMGs risk model (Figs. 1D and 2E). The equation reads as follows: risk score = ADAMTS20*(−0.864993175178699) + FREM3*(−0.405405568282815) + LAMB4*(0.994584699657483) + MMP1*(0.314532374752046) + MMP7*(0.0128808078301219).

### 3.2. Prognostic analysis and validation of the BMG risk model

**Figure 3.**
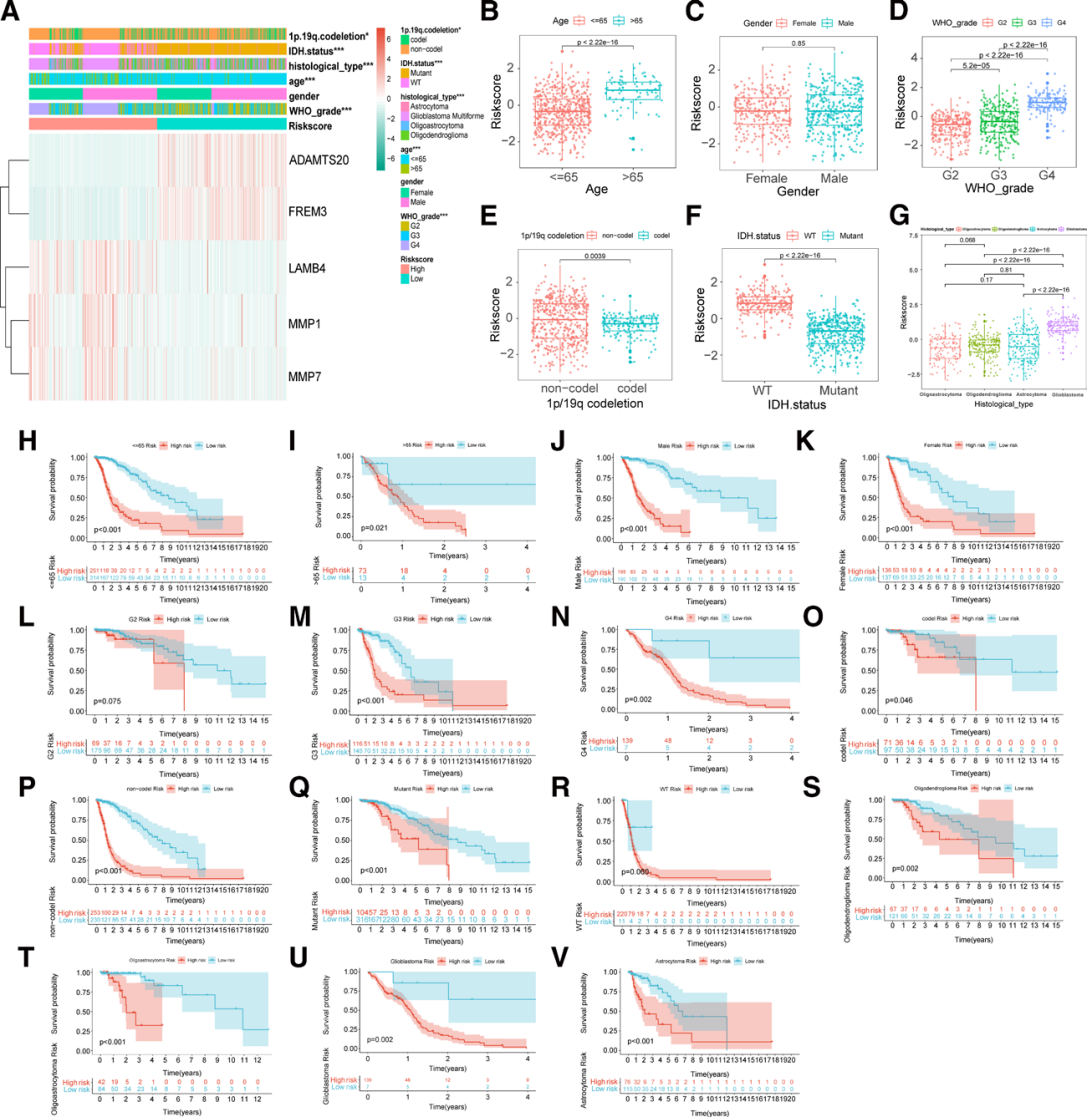
Stratified analysis of clinical characteristics in the BMGs risk model. (A) Heatmap of clinical characteristics between the high-and low-risk group. Red represents the high-risk group and green represents the low-risk group. (B–G) Differences in risk scores between subgroups of clinical characteristics, including age (B), gender (C), WHO grade (D), 1p/19q codeletion (E), IDH status (F), and histological type (G). (H–V) KM survival curves for clinical characteristics subgroups. Including <=65 (H), >65 (I), male (J), female (K), WHO G2 (L), G3 (M), G4 (N), 1p/19q codel (O), non-codel (P), IDH mutant (Q), wide-type (R), oligodendroglioma (S), oligoastrocytoma (T), glioblastoma (U), astrocytoma (V). Red represents the high-risk group and green represents the low-risk group. *P* value < .05 was deemed to be statistically significant. BMGs = basement membrane genes.

Risk profiles were performed for the training set (Fig. 3A), GEO validation set (Fig. 3B), CGGA_325 validation set (Fig. 3C), and CGGA_693 validation set (Fig. 3D). The risk scatter plots showed that BMGs risk scores in training and validation sets had favorable discrimination for survival status. The KM curve indicated that high-risk samples presented worse outcomes, and all the *P* value < .001. Using heatmap, we compared the expression of model genes in cohorts with high and low risk (Fig. 3E). The expression of ADAMTS20 and FREM3 decreased in the high-risk group, while LAMB4, MMP1, and MMP7 increased in the high-risk group (Fig. 3F), AUC at 1, 3, 5 years were all over 0.8. In GEO (Fig. 3G), CGGA_325 (Fig. 3H), and CGGA_693 (Fig. 3I) cohorts, AUC at 1, 3, and 5 years were all over 0.65. The model performed well in terms of prediction in both the training and validation sets. Univariate Cox regression suggested that WHO grades, age, histological type, IDH status, 1p.19q.codeletion, and risk score were prognostic factors for glioma (Fig. 3J). Multivariate Cox regression analysis suggested that WHO grade, age, IDH status, and risk score were independent prognostic factors for glioma (Fig. 3K). Higher WHO grades, older age, IDH wild-type, and higher risk scores are prognostic risks for patients with glioma.

### 3.3. Stratified analysis of clinical traits in the BMGs risk model

**Figure 4.**
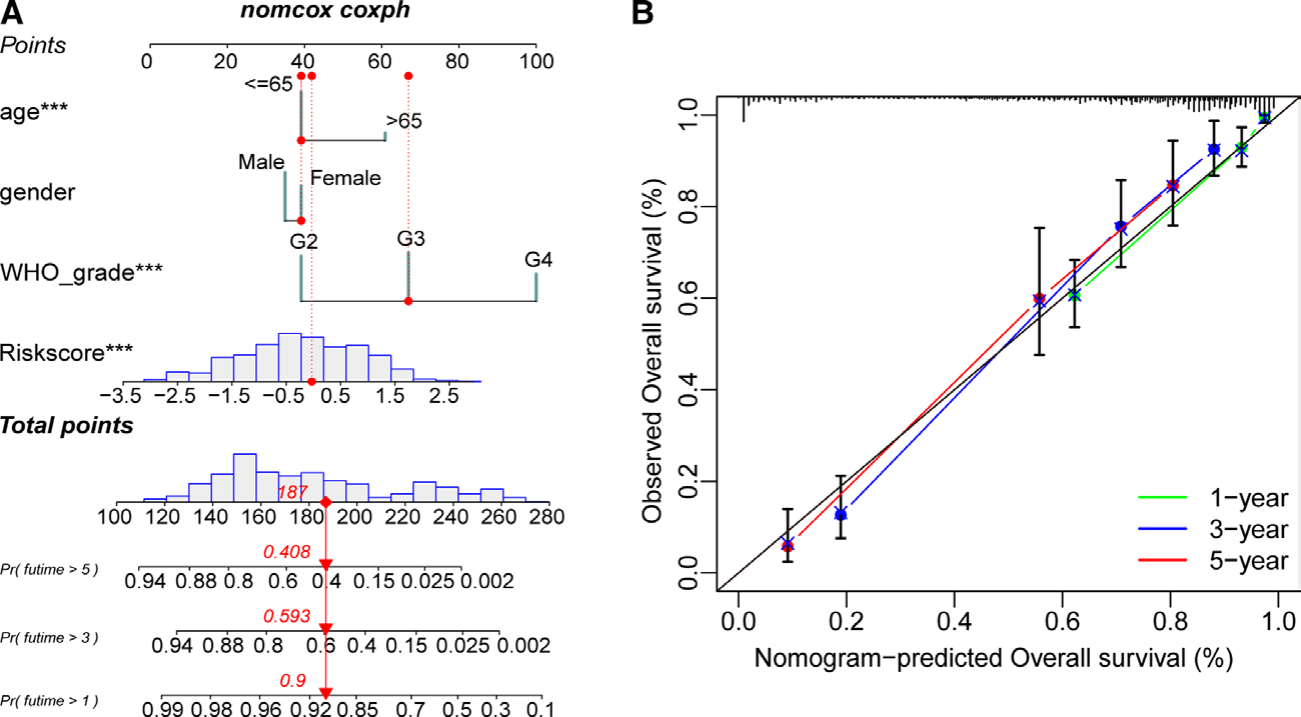
Performing and validation of nomogram. (A) Nomogram of clinical features and risk scores. *P < .05, **P < .01, ***P < .001. (B) Calibration curve of nomogram at 1, 3, and 5 yr. Green represents 1 yr, blue represents 3 yr and red represents 5 yr.

The heatmap of clinical features presented that WHO grade, age, histological type, IDH status and 1p.19q.codeletion were significantly different between high and low-risk groups (Fig. 4A). To recognize variations in clinical traits, we mapped the difference boxplots (Fig. 4B–G). The results showed that patients over 65 (Fig. 4B), with higher WHO grade (Fig. 4D), 1p/19q non-codel (Fig. 4E), IDH wide-type (Fig. 4F), and glioblastoma (Fig. 4G) had higher risk scores. We next performed subgroup KM survival analyses of clinical characteristics (Fig. 4H–V). The results indicated that all subgroups of high-risk cohorts except WHO G2 and IDH wide-type had a worse prognosis.

### 3.4. Performing nomogram and function enrichment analyses

**Figure 5.**
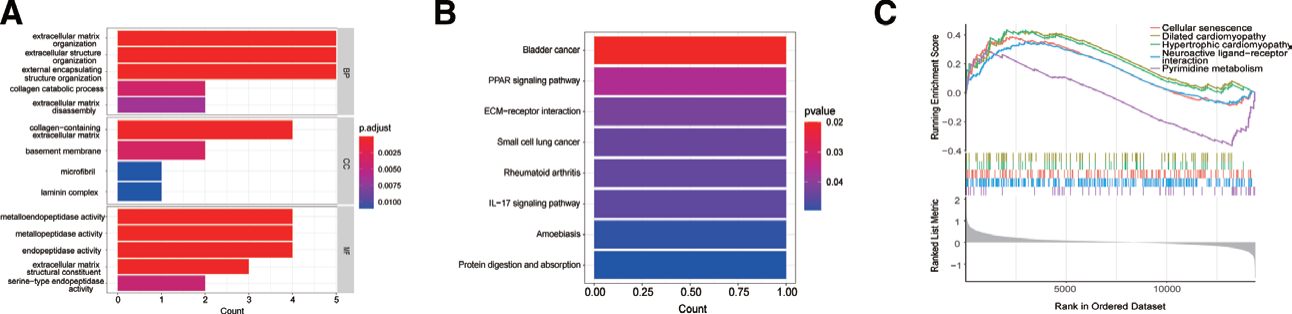
Function enrichment analyses. (A) Barplot of GO analysis. biological processes were mainly enriched in extracellular matrix organization and extracellular structure organization pathways; cellular component was mainly enriched in collagen-containing extracellular matrix and basement membrane; molecular function was mainly enriched in metalloendopeptidase activity and metallopeptidase activity. (B) Barplot of KEGG analysis. DEGs were enriched in bladder cancer, PPAR signaling pathway, and ECM-receptor interaction. (C) Visualization of GSEA analysis. Genes were enriched in cellular senescence, dilated cardiomyopathy, and hypertrophic cardiomyopathy. *P* value < .05 was deemed to be statistically significant. DEGs = differentially expressed genes, GO = gene ontology, GSEA = gene set enrichment analysis, KEGG = Kyoto encyclopedia of genes and genomes.

To facilitate the clinical application of the model, we constructed nomograms incorporating WHO grades, age, gender, and risk scores (Fig. 2A). Based on the patient clinical data and risk scores, the overall score and associated probability of survival at 1, 3, and 5 years can be determined. We assessed the predicted robustness of the nomogram at 1, 3, and 5 years using calibration curves (Fig. 2B). The nomogram had satisfactory predictive robustness at 1,3, and 5 years. For 9 DEGs, we ran a GO/KEGG functional enrichment analysis (Fig. 5A and B). In GO analysis (Fig. 5A), biological processes were enriched in pathways like extracellular matrix organization; cellular component was aggregated in pathways like collagen-containing extracellular matrix; molecular function was enriched in pathways like metallopeptidase activity. In KEGG analysis (Fig. 5B), DEGs were enriched in bladder cancer, PPAR signaling pathway, and ECM-receptor interaction. We incorporated all the resulting genes from the differential analysis into the GSEA (Fig. 5C). The main enrichments were cellular senescence, dilated cardiomyopathy, and hypertrophic cardiomyopathy.

### 3.5. Tumor immune microenvironment and drug sensitivity prediction analyses

**Figure 6.**
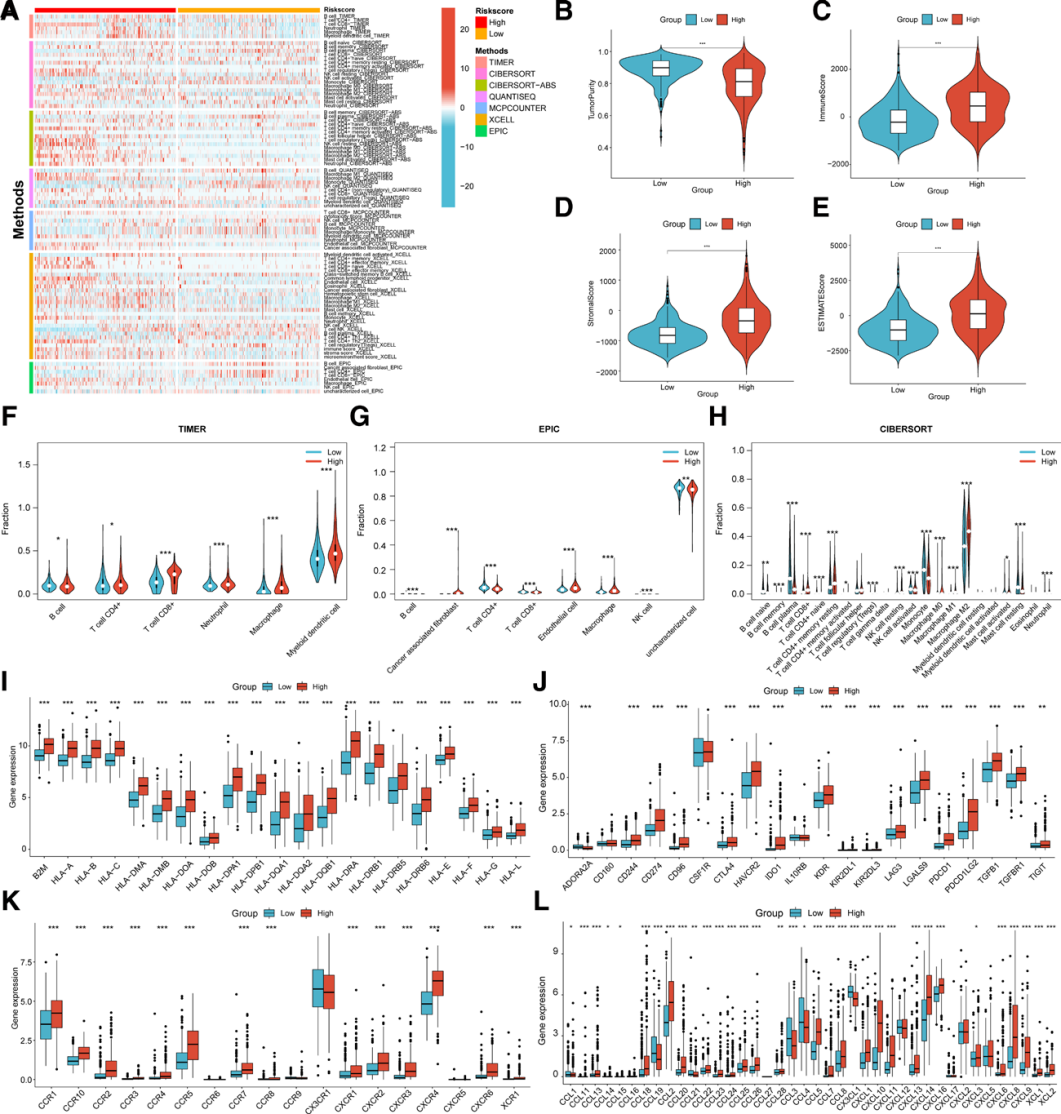
Tumor immune microenvironment analysis. (A) Immune heatmap of immune cell scores of 7 algorithms across all samples. Red represents high immune cell scores and blue represents low immune cell scores. (B–E) Boxplots of Tumor purity (B), immune score (C), stromal score (D), and ESTIMATE score (5E). (F–H) Immune cell scores boxplots of the “TIMER” (F), “EPIC” (G), and “CIBERORT” (H) 3 algorithms in the high-and low-risk groups. (I–L) Boxplots of gene expression levels of MHC molecule (I), checkpoint (J), chemokine receptor (K), and chemokine (L) between the high-and low-risk group. Red represents the high-risk group and blue represents the low-risk group. *P < .05, **P < .01, ***P < .001.

**Figure 7.**
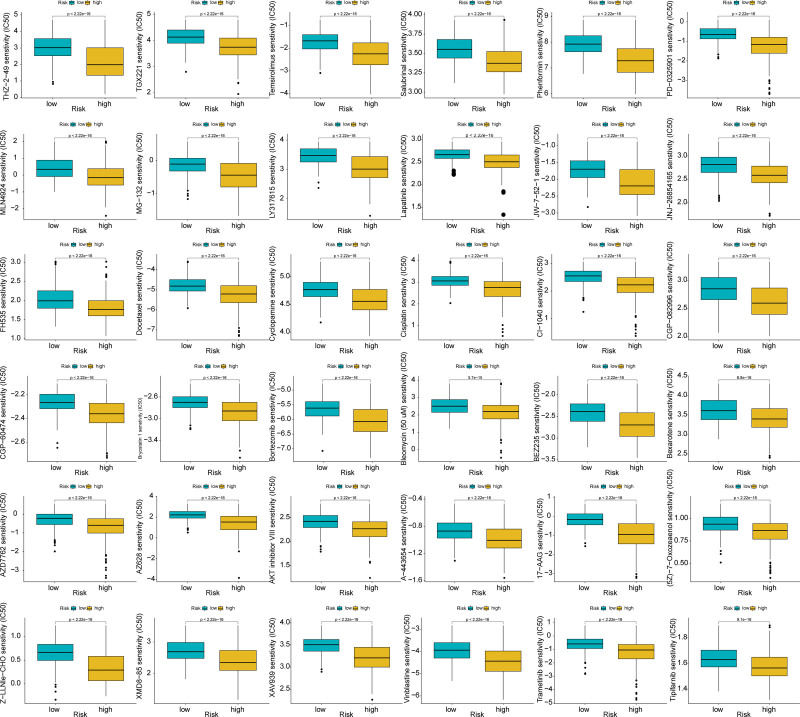
Drug sensitivity prediction analyses. Boxplot of drug sensitivity differences between high-and low-risk groups. Green represents the low-risk group and yellow represents the high-risk group. The lower value of IC50 represents more sensitivity to drugs. *P* value < .05 was deemed to be statistically significant.

The immune heatmap presented the immune cell scores of 7 algorithms across all samples, including “TIMER,” “XCELL,” “CIBERORT,” “CIBERORT-ABS,” “EPIC,” “QUANTISEQ,” and “MCPCOUNTER” (Fig. 6A). The majority of immune cell scores were greater in the group at the high-risk group. We compared the immune cell scores of the“TIMER,” “EPIC,” and “CIBERORT” 3 algorithms, and the results were shown in boxplots (Fig. 6F–H). The high-risk cohort had higher macrophage scores in all 3 algorithms. The high-risk cohort had higher neutrophil scores in “TIMER” and “CIBERORT.” The high-risk cohort showed a higher immune score (Fig. 6C), stromal score (Fig. 6D), and ESTIMATE score (Fig. 6E). In the high-risk cohort, tumor purity was lower (Fig. 6B). We compared expression levels of MHC molecule (Fig.6I), checkpoint (Fig. 6J), chemokine receptor (Fig. 6K), and chemokine (Fig. 6L) between high and low-risk cohorts. Most of the MHC molecule, checkpoint, chemokine receptor, and the high-risk cohort showed up-regulation of chemokine genes. We analyzed differences in drug sensitivity and selected 36 drug displays with significant differences in half maximal inhibitory concentration (IC50) (Fig. 7). The high-risk cohort had lower IC50, and was more sensitive to these 36 drugs.

## 4. Discussion

In the pathological process of glioma, the characteristics of tumor invasion, infiltration, and distant metastasis make the survival time and survival rate of patients worse. To elucidate the mechanisms of glioma invasion and infiltration, this study is based on the biological characteristics of BM and BMGs to clarify the role of the BMG gene in the maintenance of dryness in glioma.

For the sake of clarifying the expression of BMGs in glioma, this study screened 9 BMGs that were upregulated in glioma (FBN2, FREM3, Lamb4, MEP1A, MMP1, MMP7, OPTC, EVA1A, and ADAMTS20) from TCGA -GTEX expression matrix. We subsequently found that ADAMTS20 and FREM3 were interrelated lower risk of glioma using Cox regression analysis, whereas LAMB4, MMP1, and MMP7 showed high risk of glioma. Current studies have confirmed that MMP7 promotes the malignant progression of esophageal cancer, colon cancer, and prostate cancer.^[33–35]^ Wang et al confirmed through research that the knockout of BCAR4 inhibits the generation of MMP7 by increasing the expression of miR-227, thereby delaying the pathological process of glioma.^[36]^ And MMP7 was found to be one of the targets of miR-227 by fluorescein analysis. After overexpression of MMP7, the proliferation and invasion of glioma were enhanced. The above results confirm that MMP7 may act as a downstream target protein to promote the pathological progress of glioma. Mu et al also proved that MMP7 can affect the malignant degree of glioma as a downstream target protein.^[37]^ Zhang et al certificate that MMP1 expression increased in glioma and that knockdown of the MMP1 expression is followed by a reduction in the invasive ability of glioma.^[38]^ And MMP1 promotes the metastasis of pancreatic cancer and ovarian cancer.^[39,40]^ There is still no literature on the specific role of LAMB4 in glioma, but in gastric and colon cancers, researchers have found that LAMB4 mutations may promote the development of tumors.^[41]^ The above research shows that BMGs may be the hinge to affect the proliferation and invasion of glioma. Therefore, we constructed a BMGs-based glioma prognostic model for clinical application and conducted follow-up analyses.

We performed survival analyses in the training and validation sets and concluded that high-risk patients had poorer prognoses. Risk profile analysis showed the contribution of BMGs to glioma prognostic risk, and the model had high prognostic predictive power in glioma. Multivariate Cox regression results suggested that the results of this study were consistent with those of the above studies, indicating that this model could be used as an independent prognostic factor for glioma. Through clinical correlation analysis, this study concluded that the risk of glioma patients older than 65 years, with 1p/19q non-coding type and IDH wild-type were relatively higher. And the higher the WHO grade, especially the glioblastoma, the higher the risk score. In subgroups, the survival analysis showed that the high-risk group was related to the poor prognosis of patients (except WHO G2 and IDH wild-type). This suggests that risk scores are not perturbed by clinical characteristics and can independently predict prognosis in patients with glioma. To combine the BMG model with clinical practice, this paper constructed the nomogram. The function enrichment analysis of pathways related to BM is mainly focused on the differential genes between high-risk groups and low-risk groups, such as extracellular matrix, Laminin, and so on. This suggests further the possibility that BMGs influence glioma progression by regulating BM.

In the course of the malignant progression of glioma, the immune TME has received extensive attention. Tumor heterogeneity, drug resistance, immune escape, and other pathological characteristics affect the survival and prognosis of patients. Yu et al suggested that the current immunotherapy for glioma can be divided into 3 categories: inhibition of immune detection sites, bone marrow-targeted therapy, and vaccine.^[42]^ Sokratous et al proposed that the number of lymphocytes in glioma patients increased by 6 times after tumor vaccine inoculation, suggesting that lymphocytes may participate in the pathological changes of glioma.^[43]^ The above results confirm that TME and immune cells promote the development of glioma. The purpose of this study is to explore the effect of the immune microenvironment on glioma through immune profile analyses, and concluded that the tumor purity of the low-risk cohort was higher than that of a high-risk cohort, and the malignant degree of the tumor is negatively correlated with the purity of the tumor.^[44]^

Analysis of the immune cell score based on multiple algorithms revealed a notable increase in the macrophage score in the high-risk group, indicating that macrophages were involved in the malignant progression of glioma. In the TME, microglia, and macrophages account for about 30% to 50% of tumor tissues, and the heterogeneity of macrophages has attracted extensive attention. The high infiltration of macrophages promotes the malignant progression of the tumor, such as infiltration and invasion.^[45]^ In the TME, microglia, and macrophages are called Tumor-associated macrophage cells (TAMs), but they have specific different roles. Macrophages in a stimulated state can increase the invasiveness of tumors.^[46]^ It has been proved that macrophages and their secretions can induce the expression of MMP1 and its corresponding effects.^[47]^ Rebelo et al significantly increased the expression of MMP1 after constructing an aggressive and immune-resistant TME.^[48]^ Esra et al have shown that MMP1 and LABM4 were involved in cell proliferation, signal transduction, immune response, and other physiological processes.^[49]^ Yu et al demonstrated that TAMs and the MMP1 produced by them can shorten the cell cycle, promoting the progression and proliferation of colon cancer.^[50]^ Pyonteck et al showed that the activation of macrophages in gliomas is guided by CSF-1.^[51]^ Through the expression of BLZ945, a CSF-1R inhibitor, the volume, and weight of intracranial tumors were reduced and the survival rate was significantly improved 7 days later. Furthermore, the study showed that the suppression of macrophage by CSF-1R inhibitor could inhibit the proliferation and angiogenesis of tumor cells. These results suggest that macrophages and their derivatives in the TME can accelerate the malignant progression of glioma.

In a similar vein, the research demonstrates that patients with neutrophils have immunological scores that are higher than those of other immune cells in the high-risk group, suggesting that malignant progression of gliomas may be neutrophil involved. The neutrophil is released from the bone marrow and is involved in anti-inflammatory actions in the human body.^[52]^ It has been demonstrated that in the TME, neutrophils can promote tumorigenesis, and invasion, mediate immunosuppression, and accelerate tumor escape.^[53,54]^ According to Gabrusiewicz et al, gliomas and intracranial metastases have considerably more lymphocytes than normal.^[55]^ The research also suggested that gliomas and intracranial metastases could be identified by the neutrophil-to-lymphocyte ratio. In the immune TME of gliomas, both neutrophils and macrophages can increase the infiltration of glioma.^[56]^ Resistance to chemotherapeutic agents such as temozolomide is high in glioma with increased neutrophil, and prognosis is poor in patients with increased neutrophil and NLRS. The MMPs family participates in tumor immune escape and TME control in the immunological TME. MMP7 expression was considerably upregulated in breast cancer, according to Yuan et al.^[57]^ This study demonstrated that overexpression of YAP1/MMP7 downregulated the anti-tumor immune factors in the TME of cancer. Huang et al determined that LAMB4 is overexpressed in head and neck squamous cell carcinoma, and knockdown of LAMB4 expression can effectively delay tumor progression.^[58]^ Therefore, it can be inferred that BMGs participate in the regulation of TME, and produce corresponding pathological changes to promote tumor progression.

According to this study, the high-risk group with glioma expressed more HLA, immunological checkpoints, chemokines, and chemokine receptors. As a surface antigen of MHC, HLA plays a regulatory role in immunomodulation and response in humans. Yadav et al showed that MMP7-derived MHC-binding peptides are capable of eliciting an immune response from T lymphocytes and producing IFN-γ, which in turn can be targeted to treat tumors.^[59]^ Zhang et al concluded that the transcription of HLA-B, HLA-DPB1, and HLA-DRB1 accelerated the malignant transformation of glioma, according to Zhang et al of HLA-B, HLA-DPB1, and HLA-DRB1 accelerated the malignant change of glioma.^[60]^ By measuring HLA-DR in 60 patients with glioma, Diao et al found that glioma patients’ prognosis, survival rate, and tumor stage were all strongly correlated with HLA-DR expression.^[61]^ In the malignant progression of glioma, glioma is capable of immune evasion through immune checkpoints, thereby avoiding immune cell attacks on tumor cells. Vijver et al proved that collagen fragments secreted by MMP1 can mediate the inhibition of T cells, indirectly promoting glioma progression.^[62]^ It has been shown that the increased expression of MMP7 can shorten the cell cycle to promote tumor progression.^[63]^ And LAMB4 was positively correlated with the occurrence of colonic diverticulitis.^[64]^ Guan et al also proved that CTLA4 increased expression in glioma and mediated the invasion of glioblastoma, consistent with the conclusions of our study, therefore, immune checkpoint inhibitors can be used to effectively delay the malignant progression of tumors.^[65]^ Similarly, glioma also secretes chemokines and other substances that promote the invasive ability of glioma. Sciumè et al suggested that chemokines and their receptors secreted by glioma can promote tumor migration, proliferation, and infiltration.^[66]^ Chemokines can promote angiogenesis, and the use of chemokine antagonists, effectively prevents the proliferation of tumor cells, and delay the malignant progression. As a result, the locations of molecular targeted therapy for glioma may include HLA, immunological checkpoints, chemokines, and their receptors. Based on the above research, we performed drug sensitivity analyses and identified sensitive drugs for the high-risk group. These drugs may provide a reference for BMG-based glioma therapy.

As is well known, the malignant progression of gliomas is caused by a combination of multiple factors. Current research indicates that the malignant progression of glioma involves multiple related factors and signal transduction pathways. This article delves into the impact of BM-related genes on the malignancy and typing of gliomas at the genetic level, confirming the pathological mechanisms of BM-related high-risk genes promoting tumor immune escape and immunosuppression, and discovering the targets of related genes, suggesting that these actions sites can serve as future treatment directions for gliomas. Although the results of this study confirm at the molecular biology level that high-risk genes related to the BM are associated with the malignant progression of glioma, no relevant experimental verification has been conducted yet. In subsequent studies, we will verify the expression of the high-risk group genes mentioned above at the protein and RNA levels, and confirm the conclusion of this article by knocking down and increasing the expression of related genes through in vitro and in vivo experiments to determine changes in the proliferation, invasion, and tumor formation ability of glioma cells. Through multiple conclusions, it has been confirmed that the expression of BM-related genes, especially high-risk genes, is positively correlated with the malignant progression of glioma, providing an accurate theoretical basis for the treatment of glioma. In future research, we need to identify upstream and downstream signal transduction pathways of related genes, comprehensively understand the mechanism of action of high-risk BM-related genes in gliomas, clarify the malignant progression of gliomas, and explore methods to block the expression of genes and their related signal transduction pathways, to reduce the malignant pathological progression of gliomas, We hope to provide effective clinical treatment plans and significantly improve the prognosis of glioma patients.

In conclusion, as brain gliomas are difficult to eliminate and are likely to return after surgery, a range of programs must be used in conjunction to treat them. Furthermore, it is necessary to comprehensively understand the mechanism of malignant transformation of glioma and find reasonable therapeutic targets. This paper first elucidates the biological characteristics of BMG in the progression of glioma, and BMG can be used as a new therapeutic site. Understanding the control mechanism of gene expression in relation to the biological features of gliomas from a molecular biology perspective is crucial for the clinical treatment of gliomas.

## Author contributions

**Data curation:** Biao Yang, Xuepeng Li, Ziao Li, Jiayu Li, Xiaolong Guo, Geng Guo.

**Investigation:** Jianhang He, Zihan Zhou.

**Validation:** Xiaogang Wang, Yongqiang Wu, Wenju Zhang, Geng Guo.

**Writing – original draft:** Yanqi Sun, Ren Li, Yang Chen, Geng Guo.

**Writing – review & editing:** Yanqi Sun, Ren Li, Yang Chen, Geng Guo.
